# Identification and characterization of virulent *Aeromonas hydrophila* Ah17 from infected *Channa striata* in river Cauvery and in vitro evaluation of shrimp chitosan

**DOI:** 10.1002/fsn3.1416

**Published:** 2020-01-20

**Authors:** Vignesh Samayanpaulraj, Muthukumar Sivaramapillai, Sankara Naynar Palani, Krishnaveni Govindaraj, Vijay Velu, Uthandakalaipandian Ramesh

**Affiliations:** ^1^ Department of Molecular Biology School of Biological Sciences Madurai Kamaraj University India

**Keywords:** *Aeromonas hydrophila* Ah17, *Channa striata*, chitosan, **v**irulence factor

## Abstract

*Aeromonas hydrophila*, an inhabitant in the aquatic ecosystem is considered as an important foodborne bacterial zoonotic pathogen in aquaculture. The present study aimed to identify virulent *A. hydrophila* from naturally infected *Channa striata* in river Cauvery and in vitro evaluation of shrimp chitosan. Rimler Shotts (RS) and blood agar medium identified the presence of pathogenic *Aeromonas* sp. from the infected *C. striata*. *A. hydrophila* Ah17 was identified using 16S rRNA nucleotide sequence. Extracellular enzymes such as amylase, lipase, and protease were screened in *A. hydrophila* Ah17. Antibiotic susceptibility tests showed *A. hydrophila* Ah17 was highly resistant against *β*‐lactam, glycopeptide, macrolides, phosphonic, fucidin, and oxazolidinone classes of antibiotics. Virulent genes such as hemolysin (*aer* and *hly)*, heat‐labile enterotoxin (*act*), cytotonic heat‐stable enterotoxin (*ast*), elastase (*ahyB*), and lipase (*lip*) were identified. Growth and the viable cell population of virulent *A. hydrophila* Ah17 were significantly reduced in a dose‐dependent manner against shrimp chitosan (CHS) from *Penaeus indicus* (*P. indicus*). Thus, the present study isolated virulent *A. hydrophila* Ah17 from the environmental source and characterized in vitro with shrimp chitosan.

## Graphical abstract

Virulent Aeromonas hydrophila was isolated from the river stream and characterized by chitosan, an in vitro approach.

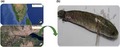

## 1 INTRODUCTION

*Channa striata,* belonging to the family Channidae*,* also known as murrel or striped snakehead fish species which are native to South Asian and African countries (Ng, 1990). It is a commercially important freshwater fish species in India, Philippines, Thailand, Cambodia, and Vietnam and accounts for 13% among the marketable freshwater fish in India (Aliyu‐Paiko, Hashim, & Shu‐Chien, 2010). According to the international union for conservation (IUCN) Red list data, *C. striata* population has been declined rapidly and it is considered under the least concern category.

The genus Aeromonads are Gram‐negative, rod‐shaped facultative anaerobe, and nonspore‐forming bacteria which are widely distributed in the aquatic environment (Daskalov, 2006). There are two groups of Aeromonads; Psychrophiles which are characterized as *Aeromonas salmonicida* and mesophilic *Aeromonas* such as *A. hydrophila* which causes the disease to warm and cold‐blooded animals (Seshadri et al., 2006). *Aeromonas* sp. have been found in surface water, groundwater, sewage, sewage effluents, and sewage‐contaminated waters (Khajanchi et al., 2010). They are clinically important due to its ability to cause septicemia, gastroenteritis, soft tissue infections, and wound to the host (Janda & Abbott, 2010).

*Aeromonas hydrophila* is a zoonotically important primary fish pathogen, which is considered as a causative agent of motile aeromonad septicaemia in freshwater fish (Chu & Lu, 2005) and eventually lead to the mortality in many fish species, particularly among South Asia's most economically important fish species *C. striata*.

The use of antibiotics for controlling *A. hydrophila* infection in farmed fish pose threats to humans and increased the incidence of antibiotic‐resistant bacteria from the environment can be observed (Yildirim‐Aksoy & Beck, 2017). Therefore, it is necessary to control bacterial disease in aquaculture in an eco‐friendly manner. Chitosan obtained by partial *N‐*deacetylation of chitin, the second most naturally available biopolymer after cellulose (Dutta, Ravikumar, & Dutta, 2002). Chitosan is extracted from the exoskeleton of crustaceans such as crab, shrimp, and lobsters, also from insects such as cockroaches, ladybird, butterfly, silkworm, and wax worm. In addition, it can be also collected from the exoskeleton of oysters, squid pen, krill, and microorganisms such as *Aspergillus niger*, *Penicillium notatum*, *P. chrysogenum,* and *Saccharomyces cerevisiae* were used as the sources of fungal chitosan (Arbia, Arbia, Adour, & Amrane, 2013). Though chitosan was obtained from the waste materials, increased attention has been made in recent times due to its biocompatibility, biodegradability, nontoxic to the biological organisms it is widely used in biomedical applications, immunological properties such as immune‐modulatory, immunoadjuvant (Zaharoff, Rogers, Hance, Schlom, & Greiner, 2007), anti‐oxidant, and antitumour (Azuma, Osaki, Minami, & Okamoto, 2015). Chitosan has been tested (in vitro) as an ideal antimicrobial agent for controlling of warm water fish bacterial pathogens (Yildirim‐Aksoy & Beck, 2017; Zheng & Zhu, 2003).

Therefore, the present study was aimed to identify and characterize the freshwater fish pathogen *A. hydrophila* from diseased *C. striata* in river Cauvery and evaluate the antimicrobial property of extracted shrimp chitosan (CHS) against virulent *A. hydrophila* Ah17.

## 2 MATERIALS AND METHODS

### 2.1 Collection of naturally infected fish

*Channa striata* displayed with the clinical signs of disease were collected from the river Cauvery, Pallipalayam, Erode District, Tamil Nadu, India (lat: 11^o^21’39.1N and long: 77^o^44’35.2E).

### 2.2 Isolation of virulent *Aeromonas hydrophila*

Ulcerated regions (skin‐lesions/muscle) of infected *C. striata* were wiped with a sterile cotton swab and suspended in physiological saline (0.85% NaCl) under aseptic conditions. The suspension was serial diluted and plated on RS agar medium (supplemented with novobiocin (5mg/L)) and incubated at 37ºC. After overnight incubation, isolates were patched on tryptic soy agar (TSA) medium for further analysis.

### 2.3 Screening of *β* hemolysin–positive isolates

For *β*‐hemolysis activity, isolates were plated on blood agar medium containing 5% (v/v) defibrinated sheep blood and the activity was observed after 24 hr at 37ºC (Santos, González, Otero, & García‐López, 1999). The *β* hemolysin–producing positive isolates were selected for further analysis.

### 2.4 PCR amplification of 16S rRNA region of *β* hemolysin–positive isolates

16S rRNA region was amplified for all the *β* hemolysin–positive isolates using the primers as described by Dorsch, Ashbolt, Cox, and Goodman (1994), and *A. hydrophila* ATCC 7966 strain was used as the positive control. Briefly, genomic DNA was extracted from *β* hemolysin–positive isolates using Bacterial Genomic DNA Purification Kit (HiMedia, Mumbai, India). Quality and quantity of genomic DNA were measured using Nanodrop™ (Thermo Fisher Scientific) and resolved using 0.7% agarose gel electrophoresis. Details of the primers and their product size are provided in Table 1. 16S rRNA gene was amplified using SureCycler 8,800 Thermal Cycler (Agilent Technologies), and the PCR product was eluted using PureLink™ Quick Gel Extraction Kit (Thermo Fisher Scientific). The eluted PCR product was cloned into TA cloning vector pXcmKn12 (Thermo Fisher Scientific) and transformed into *Escherichia coli* DH5‐*α*. Transformants were selected on Luria Bertani (LB) agar ampicillin (50 µg/ml) plate by Blue‐white selection method and confirmed by colony PCR. All the clones were sequenced in automated DNA sequencer (Xcelris Labs Limited, Ahmedabad, India).

**Table 1:** Primers used to identify *Aeromonas* sp. from the *β* hemolysin–positive isolates

Gene	Primer sequence (5’−3’)	Reference	Product size (bp)
Ah16S	*Ah16S* F‐GAAAGGTTGATGCCTAATACGTA *Ah16S* R‐CGTGCTGGCAACAAAGGACAG	Dorsch et al., 1994	686

### 2.5 Molecular evolutionary relationship of *A. hydrophila* Ah17

Similarity search was carried out for 16S rRNA nucleotide sequences of the selected isolates in nucleotide BLAST search engine tool on NCBI database (://blast.ncbi.nlm.nih.gov/). The molecular phylogenetic tree was constructed using the 16S rRNA sequence of *A. hydrophila* Ah17 (GenBank Accession no: KY646209), by Neighbor‐Joining method (Kimura, 1980) using MEGA 7 software.

The following 16S rRNA nucleotide sequences of *A. hydrophila* strains were retrieved (based on *E*‐value, Query coverage and Identity) from NCBI database; KC150866, AM992197, KC793904, JN559379, JN561162, KX980436, KX980452, KC800792, X60404, DQ095200, DQ095201, AY538658, LC200778, KC812104, KC812105, KC812106, KF146349, KF146350, EU913851, EU913854, EU913855, EU913856, HM991866, AB473043, KU570318, GU204971, KU605548, KU605549, KU605564, KU605566, KU605575, KU605578, KU605581, FN997627, KM396315. Horizontal branch lengths were drawn to scale the bar indicating 10 nucleotide replacements per site.

### 2.6 Screening of extracellular enzymes

Production of extracellular enzymes such as amylase, lipase, and protease was screened in *A. hydrophila* Ah17. Briefly, for amylase activity, the isolate was patched on starch agar medium (HiMedia) and incubated at 37ºC. After incubation, the surface of the culture was flooded with Gram's iodine (HiMedia), and appearance of the zone of clearance around the colonies was indicated as amylase‐positive isolate (Yang & Fang, 2003).

For lipase activity, the isolate was patched on tributyrin agar base (HiMedia) containing 10 ml of tributyrin and incubated at 37°C. The appearance of the zone of clearance around the colonies was indicated as lipase‐positive isolate (Collee, Duguid, Fraser, Marmion, & Simmons, 1996).

For proteolytic activity, the isolate was patched on skim milk agar (HiMedia) and incubated at 37ºC. The appearance of the zone of clearance around the colonies was indicated as protease‐positive isolate (Yang & Fang, 2003). *A. hydrophila* ATCC 7966 was used as the positive control for the study.

### 2.7 Biochemical characterization

Biochemical characterization of *A. hydrophila* Ah17 was performed by Bergey's manual of systematic bacteriology (Garrity, 2007), and *A. hydrophila* ATCC 7966 was used as the reference strain for the study.

### 2.8 Antibiotic susceptibility profile

Antibiotic susceptibility profile for *A. hydrophila* Ah17 was determined by the Kirby‐Bauer disk diffusion method (Bauer, Kirby, Sherris, & Turck, 1966). The following antibiotics were tested: Amikacin (AK: 30 μg), Amoxicillin (AMC: 30 μg), Ampicillin (AMP: 10 μg), Azithromycin (AZM: 15 μg), Cefixime (CFM: 5 μg), Cefoperazone (CPZ: 75 μg), Cefpodoxime (CPD: 10 μg), Ciprofloxacin (CIP: 5 μg), Chlorompenicol (C: 30 μg), Clarithromycin (CLR: 15 μg), Co‐Trimoxazole (COT: 25 μg), Doxycycline hydrochloride (DO: 30 μg), Fosfomycin (FO: 200 μg), Fusidic acid (FC: 10 μg), Gentamicin (GEN: 10 μg), Imipenem (IPM: 10 μg), Kanamycin (K: 30 μg), Linezolid (LZ: 30 μg), Methicillin (MET: 5 μg), Minocycline (MI: 30 μg), Nalidixic acid (NA: 30 μg), Nitrofurantoin (NIT: 300 μg), Norfloxacin (NX: 10 μg), Penicillin G (P: 10 μg), Pristinomycin (RP: 15 μg), Rifampicin (RIF: 5 μg), Streptomycin (S: 10 μg), Teicoplanin (TEI: 30 μg), Tetracycline (TE: 30 μg), Trimethoprim (TR: 5 μg), Tobramycin (TOB: 10 μg), and Vancomycin (VA: 30 μg) (HiMedia). 200 μl of *A. hydrophila* Ah17 suspension was swabbed on Mueller‐Hinton agar (MHA) medium (HiMedia) and kept for drying. Antibiotic discs were placed on the MHA medium and incubated at 37°C for 24–48 hr. The diameter of the zone of inhibition was measured, and susceptibility was categorized according to the manufacturer's protocol.

### 2.9 Identification of putative virulent factors

Virulent factors present in *A. hydrophila* Ah17 were identified using PCR‐based approach. Briefly, virulent genes such as cytolytic heat‐labile enterotoxin (*act*), cytotonic heat‐stable enterotoxin (*ast*), aerolysin (*aer*), hemolysin (*hly*), elastase (*ahyB*) and lipase (*lip*) were amplified by the respective gene‐specific primers. *A*. *hydrophila* ATCC 7966 was used as the positive control for this study***.*** Sequences of primers and the respective product sizes were provided in Table 2.

**Table 2:** Primers used for the identification of virulent genes in *A. hydrophila* Ah17

Gene	Primer sequence (5’−3’)	Reference	Product size (bp)
*hly*	*Hly* F‐ GCCGAGCGCCCAGAAGGTGAGTT *Hly* R‐ GAGCGGCTGGATGCGGTTGT	Wang et al. (2003)	130
*act*	*Act* F‐ GAGAAGGTGACCACCAAGAACA *Act* R ‐ AACTGACATCGGCCTTGAACTC	Kingombe et al. (1999)	232
*aer*	*Aer* F‐ CACAGCCAATATGTCGGTGAAG *Aer* R‐ GTCACCTTCTCGCTCAGGC	Singh, Rathore, Kapoor, Mishra, and Lakra (2008)	326
*ast*	*Ast* F‐ TCTCCATGCTTCCCTTCCACT *Ast* R‐ GTGTAGGGATTGAAGAAGCCG	Sen and Rodgers (2004)	331
*ahyB*	*Ela* F‐ ACACGGTCAAGGAGATCAAC *Ela* R‐ CGCTGGTGTTGGCCAGCAGG	Sen and Rodgers (2004)	513
*lip*	*Lip* F‐ ATCTTCTCCGACTGGTTCGG *Lip* R‐ CCGTGCCAGGACTGGGTCTT	Sen and Rodgers (2004)	382

### 2.10 Characterization of *A. hydrophila* Ah17 against shrimp chitosan (*Penaeus indicus*)

#### 2.10.1 Chitosan stock preparation

Chitosan was extracted from the exoskeleton of *Penaeus indicus* and reported in our earlier study (Samayanpaulraj, Vijay, Muthukumar, Krishnaveni, & Ramesh, 2019). Stock solution of chitosan (degree of deacetylation (DD), 84%) was prepared using 1% acetic acid and adjusted to the final concentrations of 0.05%, 0.1%, 015%, and 0.2% at pH‐6.5.

#### 2.10.2 Antimicrobial activity of shrimp chitosan against *A. hydrophila* Ah17

Antibacterial activity of CHS against *A. hydrophila* Ah17 was studied at pH‐6.5. Briefly, *A. hydrophila* Ah17 culture was taken and cells were harvested by centrifugation at 10,000 rpm for 10 min. Pellet was washed with phosphate buffer saline (PBS, pH‐7.4), and finally, the bacterial cell suspension was adjusted to 1 O.D (measured at 600 nm). 1 ml of *A. hydrophila* Ah17 cell suspension was suspended in 9 ml of CHS (0.05, 0.1, 0.15, and 0.2%) and incubated at 37°C.

Antibacterial activity of shrimp chitosan was calculated as colony‐forming unit (CFU/ml) and expressed as relative viability of *A. hydrophila* Ah17 against CHS. Briefly, bacterial culture was taken at different time intervals (0 hr, 6 hr, 12 hr, 24 hr, and 48 hr), and serial dilution was performed for all the concentrations of CHS used. Finally, 10 μl of the suspension was plated on TSA medium and incubated at 37°C. Colonies were counted at different time intervals and expressed as relative viability of *A. hydrophila* Ah17 against CHS. 1 ml of bacterial cell suspension and 9 ml of PBS were used as the control. All the experiments were performed in triplicates, and *A. hydrophila* ATCC 7966 was used as the control strain for the study.

#### 2.10.3 Bacterial cell viability assay

The relative cell viability of *A. hydrophila* Ah17 against CHS was estimated by MTT ((3‐(4,5‐dimethylthiazol‐2‐yl)‐2,5‐diphenyltetrazolium bromide) assay in 96‐well microtiter plate. Briefly, MTT (HiMedia, India) stock solution of 5 mg/ml (dissolved in PBS, pH‐7.4) was prepared and filtered through 0.22‐μm syringe filter. 1 O.D culture of *A. hydrophila* Ah17 was serially diluted in the ratio of 1:10^3^ in tryptic soy broth (TSB). 200 μl of CHS was added to the bacterial cell suspension with the final concentration of 0.05%, 0.1%, 0.15%, and 0.2% (w/v) in 96‐well microtiter plate and incubated at 37°C for 48hr. After incubation, MTT was added (0.5 mg/ml) on each well and incubated for 4.5 hr. Finally, the formazan product was solubilized by adding 100 μL of dimethyl sulphoxide (DMSO, HiMedia, India) and incubated for 30 min. After incubation, formazan product was quantified at 590 nm using microplate absorbance reader (iMark, Bio‐Rad). The quantified dissolved formazan crystals are directly proportional to the number of relative cell viability of *A. hydrophila* Ah17 against shrimp chitosan. All the experiments were performed in triplicates, and *A. hydrophila* ATCC 7966 was used as the control strain for the study.

### 2.11 Statistical analysis

One‐way analysis of variance (ANOVA) and Dunnett's multiple comparison test were performed to scrutinize the data of bacterial cell viability assay. For all comparison, *p* < .05 was considered as statistically significant. All the statistical analysis was performed using the GraphPad Prism 7.0 software.

## 3 RESULTS

### 3.1 Identification of *A. hydrophila* Ah17 strain in the river Cauvery

**Figure 1:**
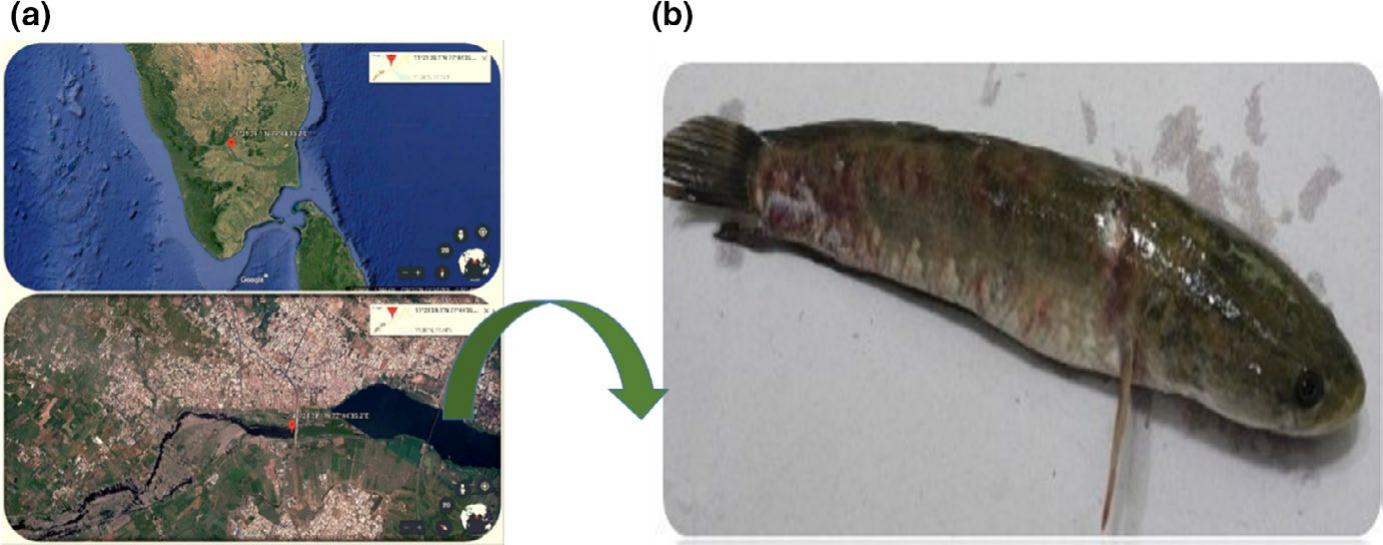
Collection site of naturally infected *C. striata*. (a) Aerial view of infected *C. striata* collection site at Pallipalayam, Erode Dt., Tamil Nadu, India (lat: 11^o^21’39.1N and long: 77^o^44’35.2E). (b) Representative image of naturally infected C. striata

**Figure 2:**
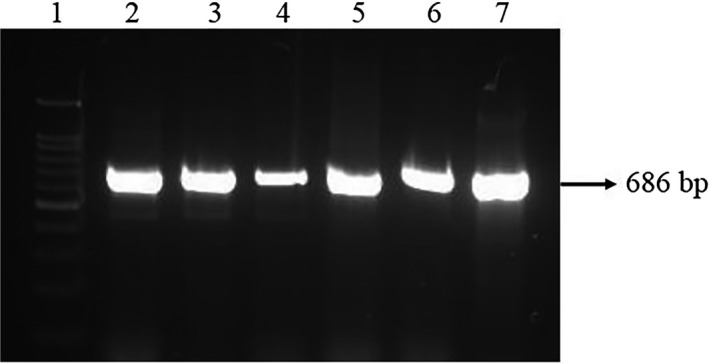
PCR amplification of Aeromonas sp.‐specific 16S rRNA region. Lane 1 represents 100bp ladder: Lanes 2–6 represent the amplification of *Aeromonas* sp.‐specific 16S rRNA region from the isolates. Lane 7 16S rRNA region from *Aeromonas hydrophila* ATCC 7966 (positive control)

Satellite view of the collection site and representative image of naturally infected *C. striata* from the river Cauvery are represented in (Figure 1a,b). A total of 430 colonies were obtained from the infected *C. striata* after screening with the RS agar medium. Among 430 colonies, 20 isolates were positive for *β*‐hemolytic activity on blood agar medium (Table 3). Out of twenty isolates (*β* hemolysin–positive isolates), five isolates were amplified (686 bp, data not shown) using *Aeromonas* sp.‐specific 16S rRNA primers (Figure 2).

**Table 3:** Observed hemolysis on blood agar media

Observed hemolysis	No. of isolates
*β*‐hemolysis	20
*α*‐hemolysis	15
*γ*‐hemolysis	25

Based on nucleotide sequencing and BLAST analysis, one isolate was identified as *A. hydrophila* (named as *A. hydrophila* Ah17 strain, Gen Bank Accession no: KY646209.1), and the rest of the isolates were identified as other *Aeromonas* sp. such as *A. veronii and A. veronii* biovar sobria.

**Figure 3:**
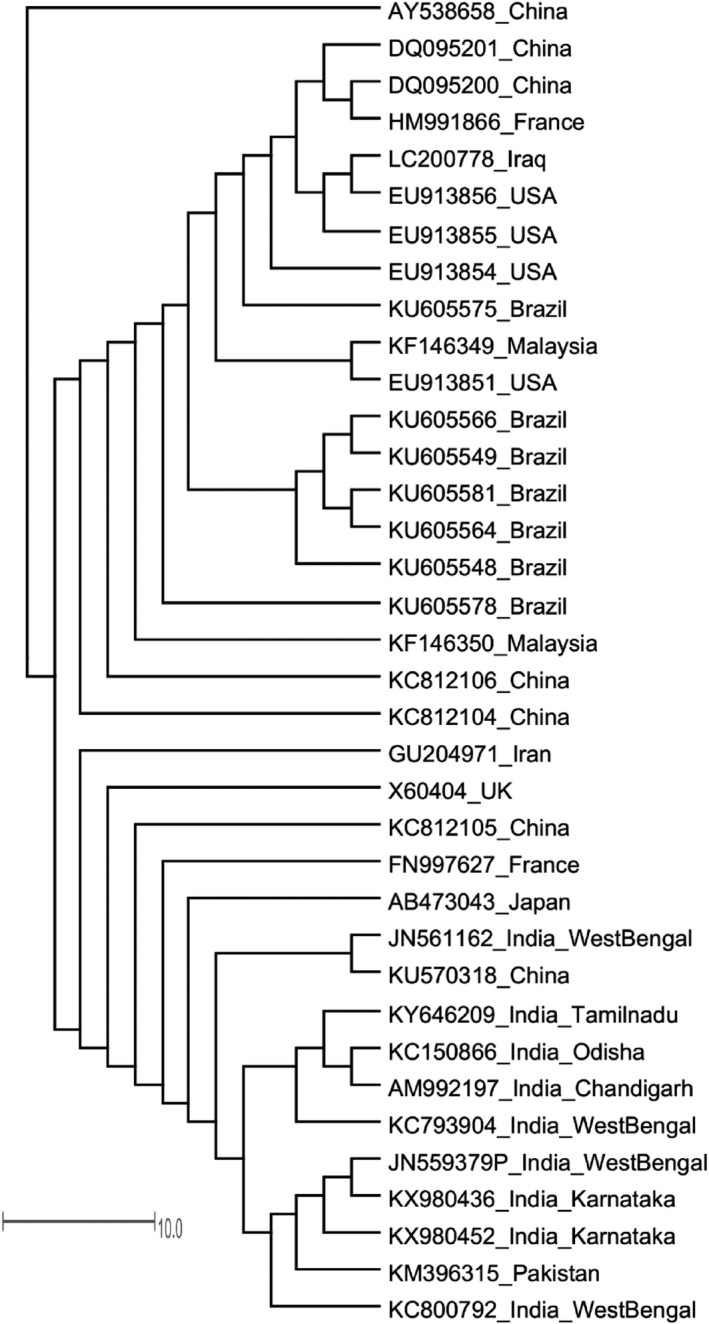
Unrooted phylogenetic tree analysis of *A. hydrophila* Ah17 by neighbor‐joining method. Scale bar represents 10 substitutions per site

Molecular evolutionary relationship studies showed that *A. hydrophila* strain Ah17 was aligned on the separate branch with KC150866 and AM992197 sequence id whereas, both KC150866 and AM992197 were found in the same clade of the Indian origin *A. hydrophila* strains. Overall, molecular evolutionary relationship of *A. hydrophila* Ah17 showed that the strain Ah17 aligned with Indian origin *A. hydrophila* strains except for JN561162 strain (Figure 3).

### 3.2 Screening of extracellular enzymes and biochemical characterization in *A. hydrophila* Ah17

**Figure 4:**
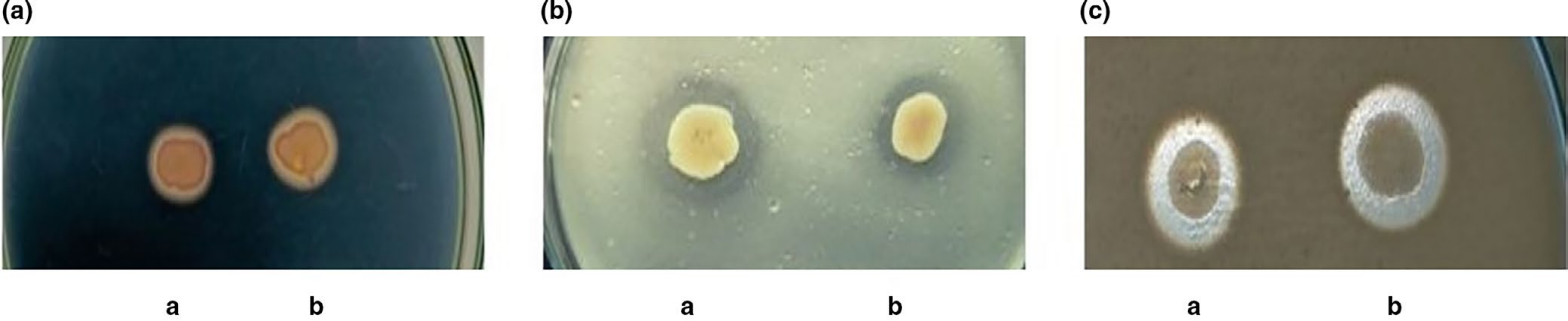
Screening of extracellular enzymes in *A. hydrophila* Ah17. (A) Starch agar medium. (B) Tributyrin agar medium. (C) Skim milk agar medium. (a) *A. hydrophila* Ah17 (b) *A. hydrophila* ATCC 7966 (positive control)

The appearance of the zone of clearance on starch, tributyrin, and skim milk agar medium indicated the production of extracellular enzymes such as amylase, lipase, and protease in *A. hydrophila* Ah17 (Figure 4). Biochemical properties such as ornithine decarboxylase were negative whereas oxidase, Voges Proskauer, motility, H_2_S production, glucose fermentation (D‐glucose), lysine decarboxylase, and arginine dihydrolase were positive for *A*. *hydrophila* Ah17 (Table 4).

**Table 4:** Biochemical characterization of *A. hydrophila* Ah17

Biochemical test	*Aeromonas hydrophila* strain	
	Ah17	ATCC 7966
Oxidase	+	+
Voges Proskauer	+	+
Motility	+	+
H_2_S production	+	+
Glucose fermentation	+	+
Ornithine decarboxylase	‐	‐
Lysine decarboxylase	+	+
Arginine dihydrolase	+	+

### 3.3 Antimicrobial susceptibility profile of *A. hydrophila* Ah17

**Figure 5:**
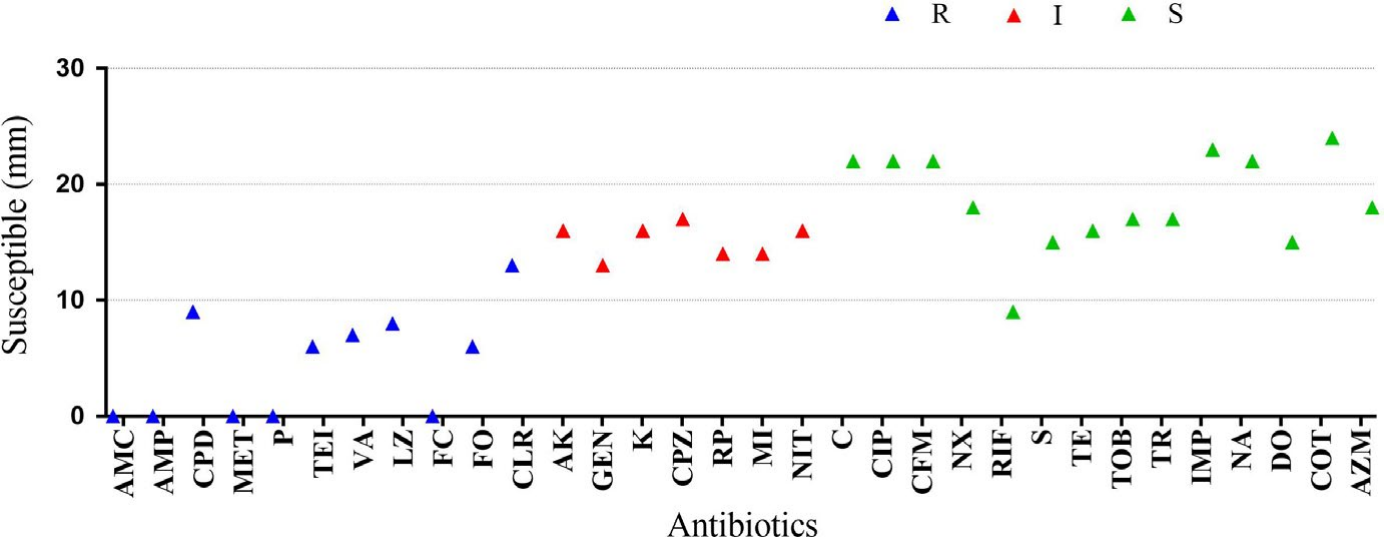
Antimicrobial susceptibility profile of *A. hydrophila* Ah17. R‐Resistance, I‐Intermediate, S‐Sensitive

Antimicrobial susceptibility profile of *A. hydrophila* Ah17 is provided in Figure 5. *A. hydrophila* Ah17 showed resistance to *β*‐lactam antibiotics such as amoxicillin, ampicillin, methicillin and penicillin G. The resistance was also observed with glycopeptide class of antibiotics (teicoplanin and vancomycin). Further, *A. hydrophila* Ah17 conferred resistance against various classes of antibiotics such as macrolides (clarithromycin), phosphonic (fosfomycin), fucidin (fusidic acid), oxazolidinone (linezolid). Aminoglycoside (amikacin, gentamicin, and kanamycin), cephalosporins (cefoperazone and cefpodoxime), streptogramins (pristinamycin), tetracycline (minocycline), and nitrofurans (nitrofurantoin) showed an intermediate response against *A. hydrophila* Ah17.

Antibiotics such as fluoroquinolones (ciprofloxacin and norfloxacin), carbapenems (Imipenem), *β*‐lactam third‐generation antibiotic cephalosporins (cefixime), aminoglycoside (streptomycin and tobramycin), tetracycline (tetracycline and doxycycline), dihydrofolate reductase (DHFR) inhibitors (trimethoprim), quinolones (nalidixic acid), rifampicin, chloramphenicol, co‐trimoxazole, and azithromycin were sensitive to *A. hydrophila* Ah17.

### 3.4 Putative virulent factors in *A. hydrophila* Ah17

**Figure 6:**
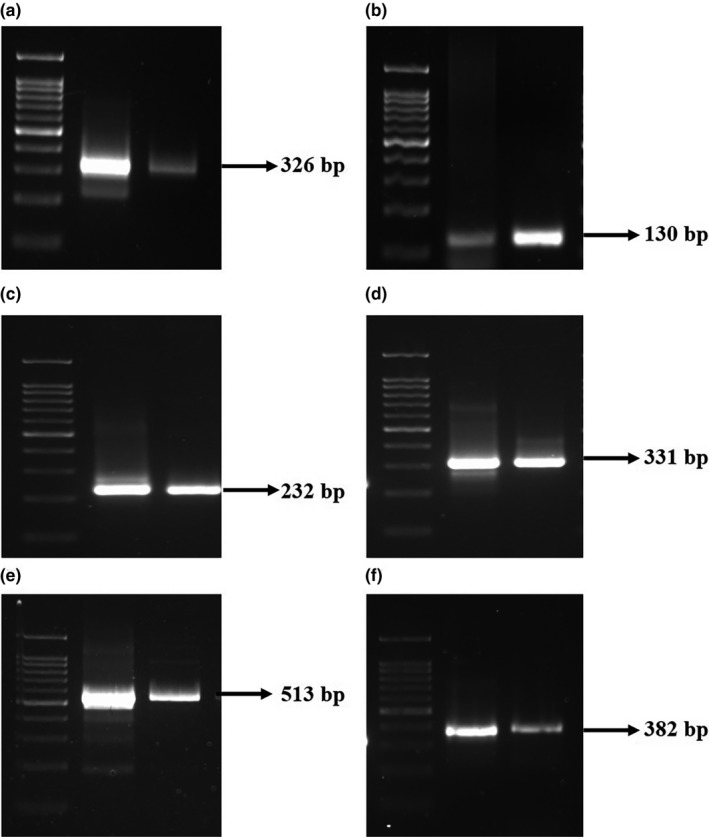
PCR amplification of putative virulent factors in *A. hydrophila* Ah17. Lane 1(a–f) represents 100bp ladder. (a) Lane 2 *aer* gene, (b) Lane 2 *hly* gene, (c) Lane 2 *act* gene, (d) Lane 2 *ast* gene, (e) Lane 2 *ahyB* gene, (f) Lane 2 *lip* gene. Lane 3 (a)‐(f) *A. hydrophila* ATCC 7966 (positive control)

PCR‐based identifications of putative virulent factors such as cytotoxins, hemolysins, lipases, and proteases were evaluated in *A. hydrophila* Ah17 (Figure 6a‐f). Since *A. hydrophila* Ah17 is a *β* hemolysin–positive strain, *aer* and *hly* genes were amplified with the product size of 326 bp and 130 bp, respectively (Figure 6a,b).

Virulent factors such as cytotoxic enterotoxin (*act*) and heat‐stable cytotonic enterotoxin (*ast*) in *A. hydrophila* Ah17 were confirmed with the isolate harbours both *act* (232 bp) and *ast* (331 bp) genes (Figure 6c,d). In addition, the presence of elastase (*ahyB*) and lipase (*lip*) genes was confirmed in *A. hydrophila* Ah17 with a product size of 513 bp and 382 bp, respectively (Figure 6e,f).

### 3.5 Characterization of *A. hydrophila* Ah17 against shrimp chitosan

**Figure 7:**
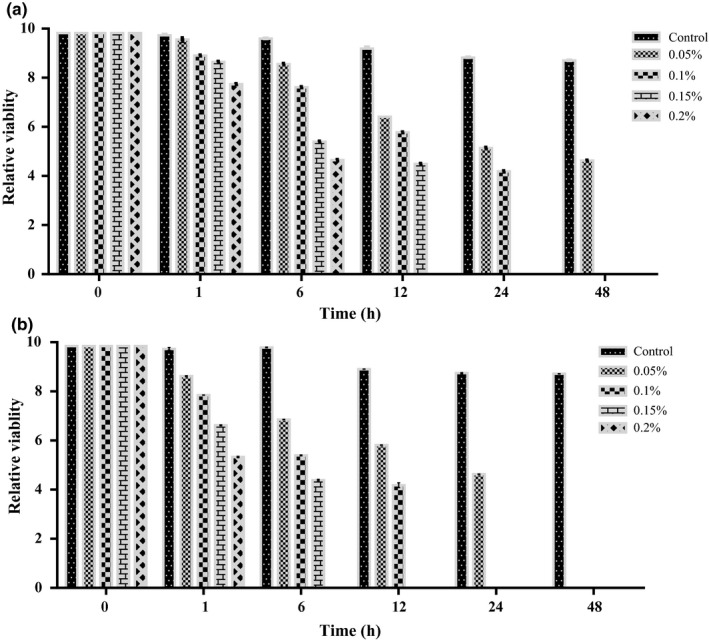
Antibacterial activity of CHS (shrimp chitosan) against *A. hydrophila* at pH‐6.5. **(**a) CHS‐treated *A. hydrophila* Ah17. (b) CHS‐treated *A. hydrophila* ATCC 7966 (positive control). Concentration of CHS (0.05, 0.1, 0.15, and 0.2%).Values are represented as mean ± *SD*

Antimicrobial activity of CHS revealed that the growth of *A. hydrophila* Ah17 was inhibited in a dose‐dependent manner at 48 hr (0.2%, 0.15%, and 0.1%), 24 hr (0.2% and 0.15%) and 12h (0.2%) (Figure 7a). On the other hand, the growth of *A. hydrophila* ATCC 7966 was inhibited at 6 hr (0.2%), 12 hr (0.2% and 0.15%), 24 hr (0.2%, 0.15%, and 0.1%), and 48 hr (0.2%, 0.15%, 0.1%, and 0.05%) in a dose‐dependent manner (Figure 7b).

**Figure 8:**
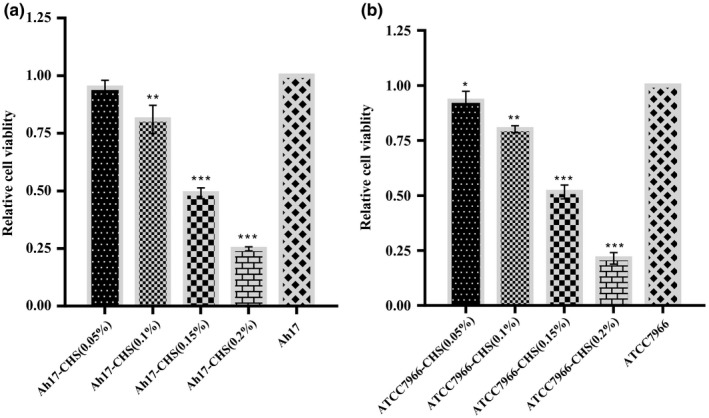
MTT assay of relative viable cell population of *A. hydrophila* **(**a) Relative viable cell population of *A. hydrophila* Ah17 treated with CHS (shrimp chitosan). (b) Relative viable cell population of *A. hydrophila* ATCC 7966 (positive control) treated with CHS (shrimp chitosan). Each bar represents the mean ± *SD*. Significant differences were observed between the test and control groups (*p < .05, **p < .01, ***p < .001) in comparison with control

The viable cell population of CHS‐treated *A. hydrophila* Ah17 group was significantly decreased in 0.2% and 0.15% (*p* < .001) and 1% (*p* < .01%) group when compared to the control group. No significant difference was observed in 0.05% CHS‐treated *A. hydrophila* Ah17 group (Figure 8a). In *A. hydrophila* ATCC 7966 group, the viable cell population was significantly decreased in 0.2% and 0.15% (*p* < .001), 0.1% (*p* < .01), and 0.05% (*p* < .5) group when compared to the control group (Figure 8b).

## 4 DISCUSSION

In recent year's great attention have been made to the genus *Aeromonas* due to its pathogenic nature in aquatic organisms as well as in humans (Furmanek‐Blaszk, 2014). Generally, it is difficult to screen pathogenic *A. hydrophila* directly from the environmental sources. Hemolytic activity is considered as one of the major characteristics property to distinguish virulent and avirulent strains in *A. hydrophila* (Wang et al., 2003). Production of the hemolytic toxin has been considered as the pathogenic potential trait in Aeromonads (Santos et al., 1999), and moreover, *β*‐hemolysin has been reported as one of the major virulent factors in motile Aeromonads (Majeed & MacRae, 1993). In line with these arguments, the identified *A. hydrophila* Ah17 strain in the river Cauvery showed characteristic *β*‐hemolytic activity in blood agar medium.

Over the last decades, strategies have been employed for the rapid and direct identification of foodborne pathogenic *A. hydrophila* strains from the environmental sources. PCR‐based microbial typing emerged as the most rapid and reliable ways to characterize and identify microbes from the environmental source (Van Belkum, Struelens, Visser, Verbrugh, & Tibayrenc, 2001). Earlier studies showed that the 16S rRNA signature sequence helps to identify *Aeromonas* sp. from the environment (Dorsch et al., 1994; Pandove, Sahota, Vikal, & Kaur, 2013). Hence, in the present study, *Aeromonas* sp.‐specific 16S rRNA oligonucleotide primers followed by nucleotide sequencing confirmed the presence of *A. hydrophila* Ah17 strain from the naturally infected *C. striata* in the river Cauvery.

With the help of molecular markers, identification of specific microbial taxa and their phylogeny was explored over several decades (Bartual et al., 2005). Among these molecular markers, 16S rRNA gene sequencing is widely used for the assessment of phylogenetic relatedness of organisms due to its functional constancy, and thus, it is considered as an effective molecular chronometer for the molecular evolutionary studies. In addition to that, it has conserved and variable regions which are evolving at a different time point that assist to measure the phylogenetic relationships. With these characteristic features, 16S rRNA region is considered as one of the useful tools for constructing evolutionary relationship at the genus level and even at the species level (Cole et al., 2008). *A. hydrophila* Ah17 is closely related to KC150866 and AM992197 and rest of the Indian isolates along with the strain KM396315 (isolated from Pakistan) formed a separate branch. Overall, all the *A. hydrophila* strains from Indian origin except JN561162 are grouped together. Thus, using 16S rRNA nucleotide sequences (based on N‐J method), evolutionary relatedness was drawn for *A. hydrophila* Ah17 strain.

Secretion system plays an important role in the pathogenicity of *A. hydrophila* into the host organism. Type II secretion system (T2SS) present in almost all members of *A. hydrophila* and it secretes extracellular enzymes such as amylase, protease etc., which contributes pathogenicity to the eukaryotic system. Earlier studies proved that highly virulent strains of *A. hydrophila* such as NJ‐35, J‐1, ML09‐119, AL09‐71 produce amylase and protease which contributes pathogenicity to the host organism (Sandkvist, 2001). In addition, extracellular lipase has been reported with virulence in many pathogens (Stehr, Kretschmar, Kröger, Hube, & Schäfer, 2003). In the present study, *A. hydrophila* Ah17 secretes amylase, lipase, and protease and it may contribute pathogenicity to the host. Ulcerative lesions and depigmentation on the caudal fins were observed during the course of *A. hydrophila* Ah17 infection in *C. striata*, which showed the pathogenic potential of the strain against freshwater fish (Samayanpaulraj, Velu, & Uthandakalaipandiyan, 2019).

Generally, *Aeromonas* sp. exhibited high resistance toward wide groups of antibiotics which are considered as the concerning factor for the treatment of *Aeromonas* infection. *A. hydrophila* Ah17 showed resistance toward most of the *β*‐lactam antibiotics except third‐generation antibiotics such as cephalosporins, cefixime which are displaying antagonistic property. It was observed that antibiotic resistance of *A. hydrophila* was mediated by chromosome‐associated *β*‐lactamse gene (Jacobs & Chenia, 2007). The resistance pattern was also observed with glycopeptide, macrolides, phosphonic, fucidin, oxazolidinone classes of antibiotics. Antibiotics resistance profile varies among strain‐specific traits such as the source of the isolates (clinical or nonclinical), aquatic environments (Henriques, Fonseca, Alves, Saavedra, & Correia, 2006), and environmental selective pressure (Janda & Abbott, 2010). Amikacin and gentamicin showed intermediate responses against *A. hydrophila* Ah17 which are in good agreement with the earlier reports (Furmanek‐Blaszk, 2014). The intermediate responses are related to uncertain susceptibility toward the therapeutic effect of antibiotics against *A. hydrophila* Ah17 (Rodloff, Bauer, Ewig, Kujath, & Müller, 2008). Thus, these variations are due to the sampling sites and type of antimicrobial agents used specifically against *A. hydrophila* infection in that vicinity (Aravena‐Roman, Inglis, Henderson, Riley, & Chang, 2012).

In general, *A. hydrophila* outbreaks are linked with the changes in host susceptibility which are caused by the environmental changes such as hypoxic conditions and increased nitrite levels in farmed fishes, as well as the significant rise in temperature. They are thought to be linked with the production of virulence factors, such as cytotoxins and hemolysins (Janda & Abbott, 2010; Mateos, Anguita, Naharro, & Paniagua, 1993).

Pathogenesis of *A. hydrophila* is multifactorial, associated with the number of virulent factors (Albert et al., 2000). PCR‐based approach identified possible virulent genes which are associated with the pathogenicity of any *A. hydrophila* strains. Therefore, in the present study, PCR‐based approaches have been carried out to detect one or more virulent genes which contribute to the pathogenicity of *A. hydrophila* Ah17, in agreement with the previous reports (Furmanek‐Blaszk, 2014; Kingombe et al., 1999; Sechi, Deriu, Falchi, Fadda, & Zanetti, 2002; Sen & Rodgers, 2004; Wang, Tyler, Munro, & Johnson, 1996). Based on epidemiological studies, the presence of these virulent factors is being used as the genetic markers to discriminate between pathogenic and nonpathogenic strains of *Aeromonas* sp. (Kingombe et al., 1999; Sen & Rodgers, 2004; Wang et al., 2003).

Virulent *A. hydrophila* produces two types of hemolysin (aerolysin, pore‐forming toxins (PFTs) and hemolysin, nonpore‐forming toxins) (Wang et al., 2003). They are the founding members of a large superfamily (β‐PTFs) that span all the kingdom of life (Szczesny et al., 2011). Studies on cryo‐electron microscopy showed that the bacterial PFTs are generally secreted as water soluble monomers and binds with target membranes and assemble into the circular oligomers, which undergoes the conformational changes that allow membrane insertion leading to pore formation and finally potential cell death (Iacovache et al., 2016). In the present study, the strain Ah17 harbors both aerolysin (*aer*) and hemolysin (*hly*) genes and the presence of these genes evidently supports the pathogenic nature of *A. hydrophila* Ah17.

Studies showed that enterotoxins, cytotoxic enterotoxin (*act*), and heat‐stable cytotonic enterotoxin (*ast*) play a major role in diarrhoeal disease (Albert et al., 2000; Sha, Kozlova, & Chopra, 2002). Besides, cytotoxic enterotoxin *act* exhibits hemolytic activity and the gene encoding these activities different from aerolysin and hemolysin (Chopra & Houston, 1999). In the present study, *act* and *ast* were identified in *A. hydrophila* Ah17 and our results are in good agreement with the earlier studies (Kingombe et al., 1999; Sen & Rodgers, 2004).

In addition to that, the presence of elastase and lipase was evaluated in *A. hydrophila* Ah17 strain which is responsible for the invasion of intestinal mucosa and establishment of the infection into the host. Mutation studies confirmed that the presence of temperature stable metalloprotease with elastolytic activity becomes more important for *A. hydrophila* virulence when tested against cold water fish *Oncorhynchus mykiss* (Cascon et al., 2000). Earlier reports confirmed that the presence of phospholipase contributes to the virulent nature of bacterial pathogens (Konig, Jaeger, Sage, Vasil, & König, 1996; Merino et al., 1999). Pathogenic strains with lipase and aerolysin genes together involved in altering the structure of the cytoplasmic membrane of the host and thereby, aggravate the pathogenic nature of *A. hydrophila* (Nawaz et al., 2010). Both *ahyB* and *lip* genes were identified in *A. hydrophila* Ah17.

Studies proved that chitosan acts as the antimicrobial agent against many foodborne pathogens such as *Candida* sp. (Rhoades & Roller, 2000), *Staphylococcus aureus*, *E· coli* (Chung, Kuo, & Chen, 2005), *Streptococcus parauberis* (Kim & Je, 2015) *Vibrio cholera* (Paredes‐Aguilar, Avila‐Sosa, & Nevárez‐Moorillón, 2017). Generally, the antimicrobial activity of chitosan depends on positive charge (polycationic amino group) on its surface molecule and these charges are mainly dependent on DD value of chitosan molecule. In the present study, CHS exhibited better antimicrobial activity and therefore inhibited the growth of virulent *A. hydrophila* Ah17 in a dose‐dependent manner. Studies proved that the growth of pathogenic *S. aureus* inhibited when DD of chitosan is high (Takahashia, Imai, Suzuki, & Sawai, 2008). Further, CHS significantly reduced the viable cell population of *A. hydrophila* Ah17 when compared to the control group. Our study is in good agreement with the study conducted by Lin, Lin, and Chen (2009) in which, viable cell populations of *A. hydrophila* were reduced at higher concentrations. Thus, the present study confirmed that the CHS with DD value of 84% showed good antimicrobial response against virulent *A. hydrophila* Ah17.

## 5 CONCLUSION

In conclusion, *A. hydrophila* Ah17 was isolated from naturally infected freshwater fish harbouring six virulent factors (*aer, hly, act, ast, ahyB,* and *lip*). In vitro characterization demonstrated that shrimp chitosan can able to control the growth of virulent *A. hydrophila* Ah17 in a dose‐dependent manner. In future, it is necessary to recognize and monitor the potential reservoirs of pathogenic bacteria and ensure their control measurements in an eco‐friendly manner, which are essentially important in epidemiological and environmental studies to prevent possible health risks.

## CONFLICTS OF INTEREST

We declare that we have no conflict of interest.

## ETHICAL STATEMENT

This study does not involve any human or animal testing.
